# A functional siRNA screen identifies RhoGTPase-associated genes involved in thrombin-induced endothelial permeability

**DOI:** 10.1371/journal.pone.0201231

**Published:** 2018-07-26

**Authors:** Joana Amado-Azevedo, Renee X. de Menezes, Geerten P. van Nieuw Amerongen, Victor W. M. van Hinsbergh, Peter L. Hordijk

**Affiliations:** 1 Department of Physiology, Amsterdam Cardiovascular Sciences, VU University Medical Center, Amsterdam, The Netherlands; 2 Department of Epidemiology and Biostatistics, VU University Medical Center, Amsterdam, The Netherlands; University of Illinois at Chicago, UNITED STATES

## Abstract

Thrombin and other inflammatory mediators may induce vascular permeability through the disruption of adherens junctions between adjacent endothelial cells. If uncontrolled, hyperpermeability leads to an impaired barrier, fluid leakage and edema, which can contribute to multi-organ failure and death. RhoGTPases control cytoskeletal dynamics, adhesion and migration and are known regulators of endothelial integrity. Knowledge of the precise role of each RhoGTPase, and their associated regulatory and effector genes, in endothelial integrity is incomplete. Using a combination of a RNAi screen with electrical impedance measurements, we quantified the effect of individually silencing 270 Rho-associated genes on the barrier function of thrombin-activated, primary endothelial cells. Known and novel RhoGTPase-associated regulators that modulate the response to thrombin were identified (RTKN, TIAM2, MLC1, ARPC1B, SEPT2, SLC9A3R1, RACGAP1, RAPGEF2, RHOD, PREX1, ARHGEF7, PLXNB2, ARHGAP45, SRGAP2, ARHGEF5). In conclusion, with this siRNA screen, we confirmed the roles of known regulators of endothelial integrity but also identified new, potential key players in thrombin-induced endothelial signaling.

## Introduction

Endothelial cells (EC), which form the inner lining of blood vessels, regulate the functioning of tissues through tight control of the extravasation of solutes, proteins and circulating cells. They are connected by tight- and adherens junctions, which are intracellularly connected to the F-actin cytoskeleton via protein complexes [1]. By rearrangement of cytoskeletal elements and actomyosin-induced contraction, EC morphology is altered, which, together with altered phosphorylation of junctional proteins, can cause either disassembly of junctions or restoration of the barrier function [2]. Vasoactive agents, such as VEGF-A, thrombin and histamine, can induce barrier dysfunction through the induction of intercellular gaps [3–6]. The consequent vascular leakage is a hallmark of many diseases and despite its importance, no therapies are available to prevent or reduce it. As RhoGTPases play an important role in the regulation of the cytoskeleton and endothelial permeability, their complex regulation may provide targets for selective improvement of a compromised endothelial barrier.

RhoGTPases are directly involved in the regulation of cell morphology, adhesion and migration and in the loss and restoration of endothelial barrier function [7, 8]. The activity of most RhoGTPases is controlled by their nucleotide (i.e. GDP or GTP)-bound state which is tightly controlled by a large number of regulatory proteins including: guanine nucleotide exchange factors (GEFs)–which promote GTPase activation by stimulating exchange of GDP for GTP; GTPase-activating proteins (GAPs)–which catalyze GTP hydrolysis; and guanine-nucleotide dissociation inhibitors (GDIs)–cytosolic chaperones which bind the bulk of inactive RhoGTPases. Atypical RhoGTPases such as RND1, RND2, RND3 [9, 10] and mitochondrial RhoT1, T2 [11, 12] are exceptions to this general process of activation, since they are not regulated by nucleotide exchange or have an extremely slow hydrolysis, keeping these GTPases constitutively active (GTP-abound). Recently, it was shown that the once considered “normal” RhoD is, in fact, also an atypical RhoGTPase [13, 14].

From the RhoGTPase family of about 20 members, Rac1 and RhoB are important regulators of the basal endothelial barrier function [15, 16]. Furthermore, RhoA received much attention, as it plays an essential role in a range of cellular processes [7, 17, 18] and in thrombin- and histamine-induced endothelial permeability [6, 19–21]. Once activated by GEFs (e.g. p115RhoGEF [22], LARG [23, 24]), RhoA signals to its downstream effector proteins, in particular RhoKinase (ROCK) which decreases the de-phosphorylation of MLC (myosin light chain) by MLC phosphatase [25]. This leads to an increase of phosphorylated MLC (phosphorylated by Ca^++^/calmodulin-activated MLC kinase) and thus enhances actomyosin-based cell contraction.

Thrombin-induced contraction and hyperpermeability are transient, and cells usually recover within 1–2 hrs from its consequences (formation of intercellular gaps, disruption of endothelial junctions) and reanneal their junctional complexes, restoring endothelial integrity. Thrombin activation also leads to a decreased activity of barrier-protecting RhoGTPases, such as Rac1 and Cdc42 [26]. FilGAP and ArhGAP22 activation by RhoA/ROCK have been suggested to play a role in the RhoA/Rac1 antagonism of tumor cells [27, 28], but inactivation of specific GEFs (p115RhoGEF, LARG, PDZ-RhoGEF) can also contribute to reduced Rac1 activation. Cdc42, on the other hand, is not inhibited but re-localized from the plasma membrane to the cytosol which leads to a delayed activation [26, 29]. Rap1 activities also contribute to the recovery phase and reannealing of junctions after thrombin-induced barrier loss [30]. Interestingly, RhoGTPases can exert both positive and negative effects on EC. Recently, RhoA was shown to be directly related to the closure of small gaps upon membrane remodeling [31] or induced by transmigrating leukocytes [32]. It appears that both localized activation of individual RhoGTPases, as well as the state of the cell prior to stimulation, determines de outcome of their signaling [33–35].

Here, we identify known and promising novel regulators of the thrombin response in endothelial cells. Using a RNAi approach in conjunction with electrical impedance measurements of the endothelial barrier, we analyzed the impact of silencing 270 genes (RhoGTPAses, RhoGEFs, RhoGAPs, GDIs, Effectors and Rho-Associated genes) on the response to thrombin. Our results confirm known regulators such as the RhoA-GEFs LARG [24] and GEF-H1 [36], the Gα protein GNA12 [37], the ROCK substrate Moesin [38, 39], and the GTPase RND3 [40]. Interestingly, our analysis also uncovers interesting, potentially novel regulators of the endothelial barrier including the GTPase RhoD, and the GEFs TIAM2, ArhGEF5, ArhGEF7 and PLXNB3.

## Materials and methods

For the stimulation with inflammatory mediators: 1% Human Serum Albumin (Sanquin, Amsterdam, The Netherlands) for 90 min and 1U/ml of Thrombin (Sigma Aldrich, Zwijndrecht, The Netherlands).

### siRNA library and functional screen

The siRNA library and screening protocol was described in our previous work [16]. Briefly, a custom ON-TARGETplus SMARTpool library (Dharmacon/GE Healthcare, Lafayette, CO) targeting 270 human RhoGTPases and Rho-associated genes (S1 Table) was used on primary human umbilical vein endothelial cells. The selection of siRNA targets was based on available literature which resulted in a total of 270 targets that represent the 6 main classes of RhoGTPases and their regulatory proteins: 82 RhoGEFs, 22 RhoGTPases, 66 RhoGAPs, 3 RhoGDIs, 21 Rho-associated proteins and 76 effector proteins. Subconfluent cells (passage 2) were seeded on 1% gelatin-coated 96W10idf arrays (Applied Biophysics, Troy, NY) in complete M199 medium (as described below). Forward transfections were performed according to manufacturer’s instructions and using siRNAs at 25nM final concentration and 0.25%(v/v) of Dharmafect 1 transfection reagent (Dharmacon/GE Healthcare, Lafayette, CO) in 100μL total volume. After 16h, medium was replaced by complete M199 medium. The screens (n = 3) were performed on different pools of HUVECs of 12 different donors. At 72h post-transfection, cells were pre-incubated with 1% Human Serum Albumin (HSA) for 90min and then stimulated with 1U/ml thrombin (more details below). siRNAs against ABL2 (#D-003101-05-0002) and OTP Non-targeting Control Pool (#D-001810-10-05) were used as positive and negative control respectively (all from Dharmacon/GE Healthcare, Lafayette, CO).

Prior to the screens, optimization experiments were carried out and transfection efficiencies were calculated by measuring mRNA knockdown of control genes (>70%). During optimization experiments the transfection efficiency of the different controls was, on average, highest at 72h post-transfection. We therefore selected this time-point for the siRNA screen.

### Endothelial cell culture

Human umbilical vein endothelial cells (HUVEC) were freshly isolated from umbilical cords of healthy donors, as previously described [3], and were obtained at the Amstelland Ziekenhuis (Amstelveen, The Netherlands). Informed consent was obtained from all donors in accordance with the institutional guidelines and the Declaration of Helsinki. The use of human tissue for isolation of endothelial cells was reviewed and approved for this study by the Medical Ethical Committee of the VU University Medical Center. After isolation, cells of different donors were pooled and resuspended in M199 medium supplemented with 100 U/mL penicillin and 100 μg/mL streptomycin, 2mmol/L L-glutamine (all Lonza, Belgium), 10% heat-inactivated human serum (Invitrogen, WI, USA), 10% heat-inactivated new-born calf serum (Lonza, Belgium), 150μg/mL crude endothelial cell growth factor (prepared from bovine brains), 5U/mL heparin (Leo Pharmaceutical Products, Breda, The Netherlands) and seeded on 1% gelatin coated plates. Cells were cultured at 37°C and 5%CO_2_ with change of medium every other day and used up to passage 2.

### Endothelial barrier function measurements

Endothelial barrier function was measured by Electrical Cell-substrate Impedance Sensing (ECIS). Briefly, passage 2 cells (1x10^4^cells/well) were seeded on 1% gelatin-coated 96w10idf arrays connected to the ECIS^®^ZTheta Array Station (Applied Biophysics, Troy, NY) and transfected as described above. Throughout the first 90h of the experiment (seeding, transfection and the following 72h), endothelial resistance data was collected by multifrequency readings of: 1000Hz, 2000Hz, 4000Hz, 8000Hz, 16000Hz, 32000Hz and 64000Hz. Barrier function assays were performed at 72h post-transfection.

For thrombin stimulation, cells were pre-incubated with M199 containing 1% HSA. After 90min of incubation, thrombin was added to the wells (1U/ml). Electrical endothelial resistance data was collected by single frequency readings of 4000Hz throughout 10h. The time-point zero in endothelial resistance graphs corresponds to the end of the 90min pre-incubation period. As control, we used NT siRNA and also silenced ABL2 gene (positive control [41] (S1D and S1E Fig).

### Area under the response curve (AUC)

Thrombin-induced responses were analyzed by real-time ECIS measurements. For each siRNA (n = 3) and NT control siRNA (n = 18, in triplicate) a period of three hours was selected for analysis that included 15min of pre-stimulation (baseline), the thrombin stimulation effect itself (drop) and the respective response to stimulation (recovery phase). Data measured on the ECIS corresponded to continuous and multiple time-point readings (~80 data-points/hour). For comparison purposes, each target siRNA and NT control siRNA response curve was standardized by dividing each value of the stimulation curve by the baseline value (15min before thrombin). AUC was calculated by means of rectangles and incorporated the entire response curve of 3h (S1A–S1C Fig; please note the color code: green = decreased AUC; blue = control thrombin response; red = increased AUC). Raw response curves of each siRNA identified as a hit can be found on supplemental data files (S2 and S3 Figs).

### Statistical analysis

Electrical endothelial resistance data was read into R [42] (version 3.4.3) and data distribution was considered to be acceptable. For a plausible comparison between different replicates (n = 3) reading values were divided by the baseline value (15min before thrombin stimulation). Comparison of each target siRNA (n = 3) and NT controls (n = 18, in triplicate) responses to thrombin stimulation was performed by two different methods. The first analysis compared 3 critical time points of the response curve: 15min before thrombin, 5min after thrombin (drop) and 2.45h after thrombin stimulation (recovery)(S1 and S2 Tables). The second approach analyzed of the complete response curve (from stimulation to recovery) by calculation of the AUC (described above) (Table 1). For each comparison, a Student’s t-test was applied, generating a single p-value for each of the 270 siRNAs, per test. The produced p-values were corrected for multiple testing by using the Benjamini-Hochberg [43] false-discovery rate (FDR). Those siRNAs with FDR<0.05 were considered significantly different from the non-targeting controls. The results presented in the manuscript are derived from the second approach, i.e. based on the AUC, as it includes the complete response (Table 1). The results of the first analysis (3 time-point comparison) are shown on supplemental tables (S1 and S2 Tables). Data is presented as mean ± SD (standard deviation).

**Table 1:** List of novel and known mediators of thrombin–induced endothelial response identified in the functional siRNA screen.

siRNA Target	AUC	2-tailed T-test p-value	FDR
RTKN	-1.267±0.04	0.00000457	0.000157
TIAM2	-1.017±0.05	0.0000116	0.001013
MLC1*	-1.145±0.02	0.0000115	0.001013
ARPC1B	-1.304±0.02	0.0000117	0.001013
SEPT2	-1.065±0.03	0.0000174	0.001097
SLC9A3R1	-0.972±0.05	0.0000191	0.001097
RACGAP1	-1.025±0.05	0.0000344	0.001686
RAPGEF2*	-1.190±0.06	0.0000508	0.001945
RHOD	-0.993±0.06	0.0001203	0.003765
PREX1*	-0.815±0.04	0.0001493	0.004281
ARHGEF7*	-1.041±0.08	0.0002136	0.004806
PLXNB2	-0.914±0.06	0.0001869	0.004806
ARHGAP45*	-0.924±0.05	0.0002235	0.004806
SRGAP2	-0.902±0.04	0.0002055	0.004806
ARHGEF5	-0.942±0.10	0.0022917	0.039419

Overview of the genes identified in the siRNA screen as significant hits and ranked by False Discovery Rate (FDR) values. Area under the response curve (AUC) determined upon stimulation with 1U/ml of thrombin (n = 3 ±SD) and T-test p-values.

*Known genes identified in this screen related to thrombin-induced signaling in endothelial cells.

## Results

### A siRNA screen identifies known and novel regulators of the thrombin-induced response in EC

To better understand the contribution of the different types of RhoGTPases to the regulation of the endothelial barrier, we performed a comprehensive RNAi screen by inducing loss-of-function of each of 270 Rho-associated genes in primary HUVEC [16] and then stimulated the monolayers with thrombin. To measure the impact of silencing of each of these genes, we analyzed the endothelial electrical impedance before, during and after stimulation. To find relevant hits, we normalized the ECIS data and determined the area under the response curve (AUC) of the response for each siRNA (n = 3) and compared it to that of the non-targeting siRNA controls (n = 18, in triplicate) (S1A–S1C Fig). Statistical analysis was performed by means of a Student’s T-test with the multiple testing correction (False Discovery Rate—FDR) set at 5%. Besides non-targeting siRNA controls, we included a siRNA targeting ABL2 as positive control (S1D and S1E Fig). This analysis resulted in a list of 15 candidate genes that included both known and novel mediators of the thrombin response in endothelial cells (Table 1). These hits are labeled by the color red and by an asterisk in the different figures of this manuscript. We noticed that the majority of these hits were genes that, at 72h post-transfection, interfered directly with the recovery of barrier function which follows the thrombin-induced loss of integrity.

To provide more insights into the genes that showed an interesting and opposite effect on the response to thrombin but did not reach statistical significance, we performed a supervised analysis and highlighted these genes in green throughout the different figures. This second analysis identified known regulators such as LARG, GEF-H1, the Gα protein GNA12, the atypical RhoGTPase RND3, and the actin regulating protein Moesin (MSN) (see below). Interestingly, this analysis also identified novel potential regulators such as the atypical RhoGTPase RhoT2 and PLXNB3.

### Silencing the atypical GTPase RhoD augments thrombin-induced endothelial barrier disruption

**Fig 1:**
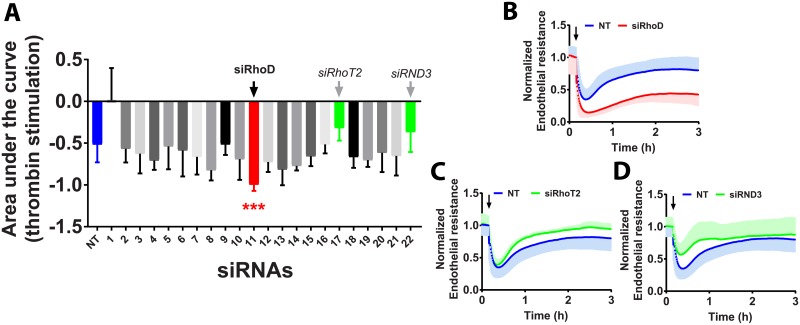
RhoGTPases and thrombin stimulation. A—Quantification of the area under the response curve (AUC) of HUVECs transfected with 22 different RhoGTPase-targeting siRNAs and stimulated with 1U/ml of thrombin for 3h at 72h post-transfection (n = 3±SD). In blue, left, NT control siRNA (n = 18, in triplicate), in red, siRhoD significantly different AUC compared to NT control siRNA and in green, two potentially relevant genes: siRhoT2 and siRND3. (Color code refers to the change in AUC, see S1A–S1C Fig) ***P<0.001 in two-tailed Student’s T-test and FDR<0.05; B-D—Normalized endothelial resistance of HUVECs transfected with indicated siRNAs and stimulated with 1U/ml of thrombin for 3h at 72h post-transfection (n = 3±SD). Arrow indicates the time point at which thrombin was added.

Among all 22 known RhoGTPases analyzed (Fig 1A) which included RhoA, Rac1 and Cdc42, loss of RhoD resulted in the strongest potentiation of thrombin-induced disruption of endothelial barrier integrity (Fig 1B). The AUC of the thrombin response of EC lacking RhoD is almost double that of NT control siRNA transfected cells. Moreover, we observed that silencing RhoD in HUVECs decreased slightly the basal endothelial barrier resistance (~30%) when compared to NT control siRNA transfected cells (S2A Fig). Finally, RhoD is required for the recovery after thrombin-treatment. This suggests an additional function for RhoD in the regulation of endothelial integrity.

### The atypical RhoGTPase RND3 and mitochondrial RhoGTPase2 (RhoT2) differentially modulate the response to thrombin

HUVECs transfected with either siRhoT2 and siRND3 show an increased basal endothelial barrier resistance when compared to NT control siRNA transfected cells (Fig 1C and 1D; S2B and S2C Fig). Interestingly, upon stimulation with thrombin, loss of RhoT2 improves the recovery but does not affect maximal thrombin-induced contraction and loss of barrier integrity, consistent with the notion that these are discrete phases with differential regulation [44]. Loss of RND3, on the other hand, attenuates and improves the thrombin-induced decrease of barrier integrity (Fig 1A and 1D), possibly related to its ability to induce stress fibers in endothelial cells through RhoB [45].

### Downregulation of TIAM2, RAPGEF2, PREX-1, ArhGEF7 and ArhGEF5 negatively affect the endothelial barrier after thrombin stimulation

**Fig 2:**
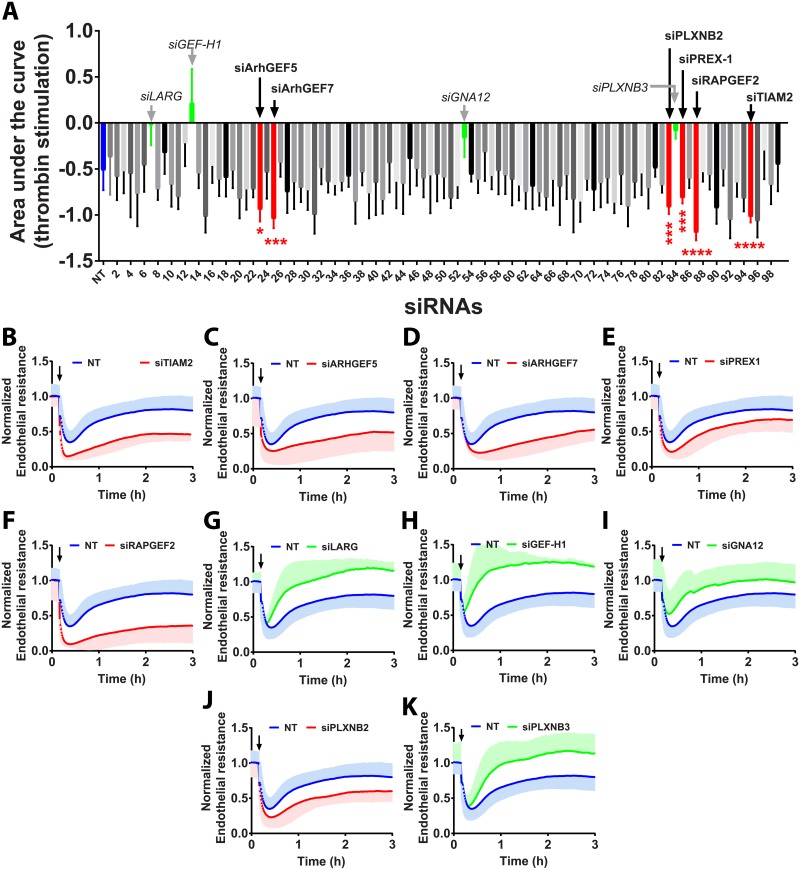
Contribution of RhoGEFs and associated proteins to the response to thrombin. A—Quantification of the area under the response curve (AUC) of HUVECs transfected with 99 different siRNAs targeting RhoGEFs (and associated genes) and stimulated with 1U/ml of thrombin for 3h at 72h post-transfection (n = 3±SD). In blue, on the left, NT control siRNA (n = 18, in triplicate), in red, RhoGEFs significantly different AUC compared to NT control siRNA and in green: siLARG, siGEF-H1, siGNA12 and siPLXNB3. (Color code refers to the change in AUC, see S1A–S1C Fig) ****P<0.0001 in two-tailed Student’s T-test and FDR<0.05; ***P<0.001 in two-tailed Student’s T-test and FDR<0.05; **P<0.01 in two-tailed Student’s T-test and FDR<0.05; *P<0.05 in two-tailed Student’s T-test and FDR<0.05; B-K, Normalized endothelial resistance of HUVECs transfected with the indicated siRNAs and stimulated with 1U/ml of thrombin for 3h at 72h post-transfection (n = 3±SD); Arrow indicates the time point at which thrombin was added.

GEFs are crucial activators and regulators of RhoGTPase activity. However, not all GEFs are involved in the response to vaso-active mediators such as thrombin (Fig 2A). Silencing these five GEFs: ArhGEF5, ArhGEF7, PREX-1, RAPGEF2 and TIAM2, caused a significant increase in the thrombin-induced contraction and delay of subsequent recovery (Fig 2B–2G). Knock-down of TIAM2, ArhGEF5 and ArhGEF7 induced a 2-fold, significant increase in AUC compared to NT control siRNA. Interestingly, however, knock-down of ArhGEF5 did not affect basal endothelial resistance (S2D Fig) whereas lack of ArhGEF7 and PREX1 in primary endothelial cells promoted an increase in basal endothelial barrier resistance (~25% and ~20% respectively) (S2F and S2G Fig). Absence of RAPGEF2 (also known as PDZGEF2) induced the most striking impact on the response to thrombin (Fig 2F), including a steep drop of resistance followed by a blunt delay in recovery after thrombin stimulation. No effects on basal endothelial resistance were observed (S2H Fig). Thus, these GEFs all play a positive role in the control of endothelial barrier function, albeit that for some, this only becomes apparent during the recovery phase following stimulus-induced barrier loss.

Loss of the GEFs LARG (Fig 2G) and GEF-H1 (Fig 2H), despite not reaching statistically significant effects, did show a marked attenuation of the response to thrombin. This is well in line with the fact that both are RhoA regulators [23, 46] and the notion that loss of RhoA signaling impairs the thrombin-induced barrier loss [20, 36]. The same appears to hold for the GNA12 protein, (Fig 2I) albeit to a lesser extent. Loss of GEF-H1 did not affect the basal endothelial resistance (S2I Fig) while loss of LARG (S2J Fig) and of GNA12 (S2K Fig) induced a small increase in basal endothelial resistance.

### PLXNB3 is a novel regulator of the thrombin-mediated response in EC

Plexins are known receptors for semaphorins which control RhoGTPase signaling [47]. Silencing PLXNB2 in HUVECs and stimulating them with thrombin induced a significant increase in the AUC which correlates directly with an impaired recovery (Fig 2J). In marked contrast, loss of its homolog PLXNB3 attenuated considerably the thrombin-induced response and induced a faster recovery (Fig 2K) despite not reaching statistical significance. Silencing PLXNB2 also increased significantly basal barrier resistance (S2L Fig) when compared to NT control siRNA whereas lack of PLXNB3 did not affect basal endothelial resistance (S2M Fig). These opposing results hint towards differential substrate specificity among these Plexin isoforms.

### ArhGAP45, RacGAP1 and SRGAP2 play an important role during the recovery phase

**Fig 3:**
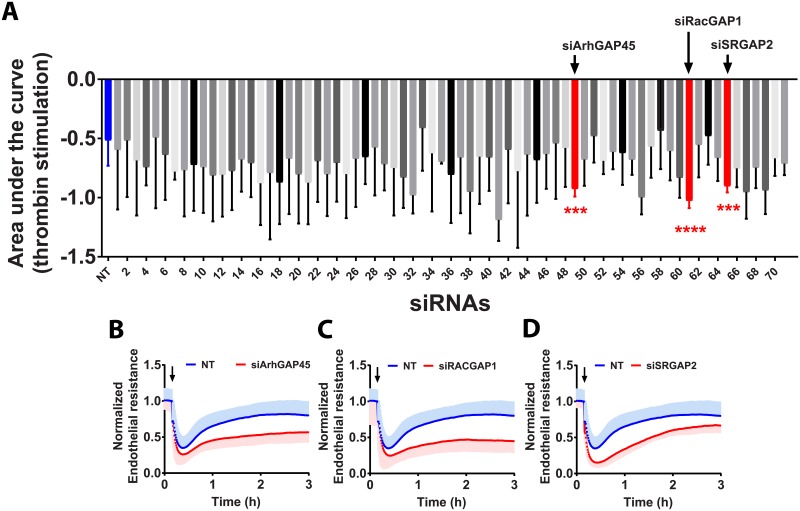
Contribution of RhoGAPs and associated proteins to the response to thrombin. A—Quantification of the area under the response curve (AUC) of HUVECs transfected with 71 different siRNAs targeting RhoGAPs (and associated genes) and stimulated with 1U/ml of thrombin for 3h at 72h post-transfection (n = 3±SD). In blue, on the left, NT control siRNA (n = 18, in triplicate), in red, RhoGAPs significantly different AUC compared to NT control. (Color code refers to the change in AUC, see S1A–S1C Fig) ****P<0.0001 in two-tailed Student’s T-test and FDR<0.05; ***P<0.001 in two-tailed Student’s T-test and FDR<0.05; B-D—Normalized endothelial resistance of HUVECs transfected with the indicated siRNAs and stimulated with 1U/ml of thrombin for 3h at 72h post-transfection (n = 3±SD); Arrow indicates the time point at which thrombin was added.

RhoGAPs are key regulators of GTPase activities. Out of the 71 GAPs analyzed in our screen, three showed significant effects on the thrombin-induced effects on the endothelium (Fig 3A). Upon thrombin stimulation, lack of these genes (ArhGAP45, RacGAP1 and SRGAP2) affected both the reduction in electrical resistance and the subsequent recovery (Fig 3B–3D). Silencing ArhGAP45, RacGAP1 and SRGAP2 also increased basal barrier resistance in HUVECs (~25%) when compared to NT control siRNA transfected cells (S2N, S2O and S3A Figs). In contrast to our findings for the GEFs, we did not find GAPs of which their loss promotes endothelial barrier function. Clearly, ArhGAP45, RacGAP1 and SRGAP2 appear all required for a full recovery after thrombin-induced loss of integrity.

### Loss of the effector proteins RTKN, ARPC1B, MLC1, SETP2 and SLC9A3R1 potentiates the thrombin response

**Fig 4:**
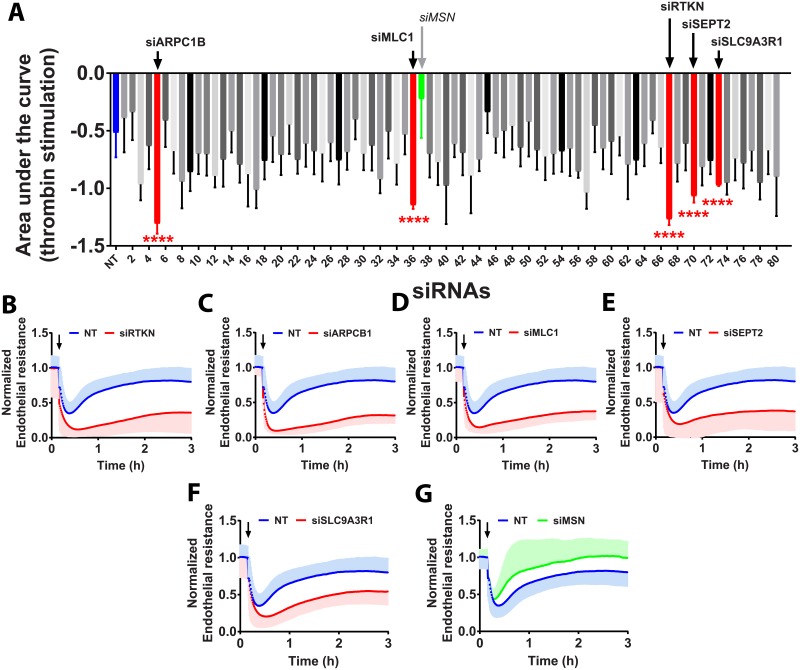
Contribution of Rho effector proteins and associated proteins to the response to thrombin. A—Quantification of the area under the response curve (AUC) of HUVECs transfected with 80 different siRNAs targeting Rho-effector proteins (and associated genes) stimulated with 1U/ml of thrombin for 3h at 72h post-transfection (n = 3±SD). In blue, on the left, NT control siRNA (n = 18, in triplicate), in red Rho-effector proteins with a significantly different AUC compared to NT control siRNA and in green, siMLC1. (Color code refers to the change in AUC, see S1A–S1C Fig) ****P<0.0001 in two-tailed Student’s T-test and FDR<0.05; B-G—Normalized endothelial resistance of HUVECs transfected with the indicated siRNAs and stimulated with 1U/ml of thrombin for 3h at 72h post-transfection (n = 3±SD); Arrow indicates the moment at which thrombin was added.

Out of 81 Rho-effector proteins and other Rho-associated genes analyzed, five showed significant effects on endothelial barrier integrity (Fig 4A). Silencing the effector proteins Rhotekin (RTKN) and Actin Related Protein 2/3 Complex Subunit 1B (ARPC1B) increased basal endothelial barrier resistance (S3B and S3C Fig) although it also increased, in an opposite way, the AUC almost 3-fold (Fig 4B and 4C). This is due to an increase in the loss of electrical resistance upon stimulation with thrombin, followed by a slow and inefficient recovery phase. This phenotype can also be observed by downregulation of myosin light-chain 1 (MLC1) or Septin2 (SEPT2), albeit to a lesser extent (Fig 4D and 4E). No changes in basal endothelial barrier resistance were observed for MLC1 or SEPT2 (S3D and S3E Fig). Silencing Solute Carrier Family 9 Isoform A3 Regulatory Factor 1 (SLC9A3R1) increased the basal endothelial barrier resistance (S3F Fig) and induced a significant (2-fold) increase in the AUC when compared to the NT siRNA controls (Fig 4F).

Silencing the effector protein Moesin (MSN) showed an attenuation of the thrombin-induced drop in resistance followed by a steeper and faster recovery (Fig 4G) and no impact on basal endothelial barrier resistance (S3G Fig). Thus, of these effector proteins, only Moesin appears to play a role in barrier disruption.

## Discussion

The functional connection between endothelial activators and control of vascular permeability has been widely studied over the past decades but remains incompletely understood. Activation of the RhoGTPase RhoA became a central event in studies of thrombin-induced permeability and the associated signaling pathway was characterized in considerable detail [3, 20, 48–51]. However, the RhoGTPases and their regulators have not been analyzed simultaneously in a comprehensive screen. The current study aimed to improve our understanding of the relevant mechanisms and to identify novel molecular players in the control of endothelial integrity.

Recently, we published the first results of this screen, focusing on the control of basal endothelial integrity [16]. We identified RhoB- (negative regulator) and Cdc42- (positive regulator) associated signaling pathways which control the dynamics of endothelial integrity in resting monolayers. Here, we extend this analysis, focusing on agonist-induced loss and recovery of endothelial cell-cell contact, using the protease thrombin as a strong activator of the endothelium. Under the described conditions and at 72h post siRNA transfection, our analysis identified 15 different proteins that play a role in the thrombin-induced response of endothelial cells (RTKN, TIAM2, MLC1, ARPCB1, SEPT2, SLC9A3R1, RACGAP1, RAPGEF2, RHOD, PREX1, ARHGEF7, PLXNB2, ARHGAP45, SRGAP2, ARHGEF5). Loss of these 15 genes resulted in an increased contraction and delayed or impaired recovery phase post-thrombin stimulation. Moreover, based on a supervised analysis of the response curves we identified seven additional proteins with opposite effects to the ones just described, i.e., by being silenced, these proteins attenuated contraction or improved recovery. Of these seven negative regulators, two are new potential regulators (RhoT2 and PLXNB3) and five are previously described permeability mediators (RND3, GEF-H1, LARG, GNA12 and MSN). In this discussion, we focus on the most significant and new hits form this screen.

The current study has primarily identified proteins, the loss of which augments thrombin-induced decrease of barrier function and subsequent recovery. This suggests that endothelial cells express more barrier-protective than barrier-disruptive RhoGTPase-related proteins. This is physiologically relevant, as the endothelial barrier is essential for the circulation and for normal functioning of tissues and organs. Thus, loss of integrity should be carefully regulated and sufficiently limited to prevent edema, inflammation and tissue damage. Although this notion would predict considerable redundancy, we were able to identify a sizeable number of key regulatory proteins suggesting that these play unique roles in barrier-regulating signaling pathways. So, although endothelial integrity is protected by many proteins at different levels, it is also vulnerable, since the associated redundancy appears limited. Moreover, protein turnover and expression levels in HUVECs must be taken into consideration. Like with any siRNA screen, the chosen time points are based on average knock-down efficiency of controls usually determined during optimization experiments and not on individual siRNAs includes in the screen. Thus, we cannot exclude that some of the targeted proteins have already partially restored expression in the course of the experiment.

Thrombin activates the PAR-1 receptor which, through various G-proteins (Gα_11/Q_, Gα_12/13_ and G_αi_), will lead to activation of RhoA. Our screen confirms that the Gα12 subunit plays a crucial, protective role because lack of this gene attenuates thrombin-induced permeability [37]. Downstream of Gα12, the RhoGEFs LARG and GEF-H1 control endothelial integrity, as demonstrated previously [24, 36, 52]. According to our analysis, no other tested GEFs have similar effects, although perhaps not all of these GEFs are expressed in ECs [53].

Our findings further show that loss of RAPGEF2 (aka PDZGEF-1) or ArhGEF5 (aka TIM) does not affect the integrity of the barrier under basal conditions but both enhance the maximal barrier-disruptive effect of thrombin and reduce the subsequent recovery. ArhGEF5/TIM can activate RhoA and RhoB in HEK293 cells [54] but also Rac1 and Cdc42 [55], making its precise function in endothelium as yet unclear. Interestingly, RAPGEF2/PDZ-GEF1 is a Rap1-specific GEF which positively controls endothelial junctions in conjunction with EPAC1 [56]. This suggests that in primary endothelial cells, PDZGEF-1 and ArhGEF5 most probably act as GEFs for barrier-enhancing GTPases such as Rac1, Cdc42 or Rap1. PREX-1 and ArhGEF7 (aka betaPIX) were other GEFs identified in our screen that induced significant differences in the response to thrombin. Both GEFs are claimed to be specific for RAC1 [57, 58] and our data shows that loss of these GEFs leads to a delayed recovery of the endothelial barrier, supporting the notion that these are Rac1-activating GEFs.

Unexpectedly, loss of RhoA, Rac1 or Cdc42 did not induce significant differences in the response to thrombin when AUC was analyzed. In line with a recent study from our group [44] we showed that loss of RhoA increases expression of RhoC and, slightly, of RhoB, suggesting that a shift in balance among RhoGTPases influences their contribution to thrombin-induced barrier loss. Moreover, we also showed recently that thrombin can activate RhoA, RhoB as well as RhoC with similar kinetics [59]. Surprisingly, the atypical GTPase RhoD appears to play a barrier-protective role. This is in line with recent data showing that RhoD stabilizes the actin cytoskeleton [13] and inhibits RhoC-ROCK-dependent cell contraction [60].

Once activated, RhoGTPases can interact with several downstream effectors which are directly and indirectly associated with (regulators of) cytoskeletal elements and junctional proteins. Interestingly, most of the ‘effector’ hits identified in our screen play a barrier-protective role, in particular during the recovery phase following thrombin stimulation. For ARPCB1 this is in line with its role in the Arp2/3 complex, which is activated by Rac1 and is required for membrane protrusion and formation of cell-cell contacts [61]. MLC1 on the other hand would be expected to be barrier disruptive due to its role in contractility, similar to the GTPase-binding protein SEPT2 which was previously implicated in cell division and associated with actin fibers [62]. Apparently, MLC1 in EC also plays a role in barrier restoration, possibly by formation of contractile actin bundles, parallel to cell-cell contacts. Similarly, SLC9A3R1, also known as ERM binding protein 50 (EBP50) or NHERF1 (Na+/H+ exchange regulatory factor) is a PDZ-domain containing scaffolding protein that is implicated in cancer, cell polarity and actin regulation [63, 64]. In the vasculature, SLC9A3R1 promotes neointima formation following arterial injury while in vascular smooth muscle cells, and promotes focal adhesion turnover and migration [65]. Intriguingly, SLC9A3R1 binds to β-catenin and was found to stabilize β-catenin-E-cadherin complexes [66], which is in line with our findings regarding its positive role in endothelial integrity.

Plexins are important cell-surface receptors for semaphorins and are involved in RhoGTPase signaling, acting as GAPs [47, 67, 68]. Our findings show that silencing PLXNB2 promotes both a significant increase of basal barrier resistance [16] and a significantly augmented response to thrombin. PlexinB2 negatively regulates Rac1 and Cdc42 [69] which is in line with our findings but is in contrast to the effects of PLXNB3. Recently, it was shown that all three plexin-B proteins interact with RAP1 and that PLXNB2 interacts with RND3 [70, 71]. Our data support the possibility that PLXNB3 acts as a GAP for RAP1 as loss of this gene promotes enhanced recovery post-thrombin, while our findings suggest that PLXNB2 may have GAP activity towards barrier promoter RhoGTPases such as Rac1 and Cdc42.

Several other RhoGAPs have previously been implicated in endothelial cell functions, such as p190RhoGAP, ArhGAP22 and FilGAP. However, downregulation of these GAPs did not significantly alter the response to thrombin in our screen. We did identify ArhGAP45, aka HMHA1 [72], RACGAP1 and SRGAP2 which were not previously linked to thrombin-induced permeability but are clearly required for barrier restoration. Downregulation of these GAP proteins increases endothelial basal barrier resistance which is line with other studies linking them to Rac1 activity, including our work on ArhGAP45. Surprisingly, loss of any of these GAPs impairs contraction or enhances barrier restoration following thrombin stimulation despite the Rac1 inactivation being absent. The mechanistic explanation for this is unknown, although our previous work indicated that the imbalance between Rac1 vs RhoA-induced signaling, which results from GAP-mediated downregulation of GTPase activity, may impair restoration of cell-cell contacts. However, this phenomenon requires additional study.

In summary, under the conditions described in this manuscript, we identified potential novel regulators of thrombin-regulated endothelial permeability, the majority of which appear to be barrier-protective. Out of 270 genes analyzed, loss of 15 induced a significant change to the effect of thrombin, comprising transient contraction and full barrier restoration. Our findings provide new insights on previously unidentified regulators that play a key role in thrombin-induced contraction or the subsequent junctional recovery phase. Future research may reveal if any of the identified proteins represents potentially new targets for treatments aimed at preserving vascular integrity.

## Supporting information

S1 Fig Area under the response curve and controls. A-C—Schematic representation of area under the response curve (AUC) interpretation. Color code refers to the changes in AUC, i.e., **blue** reflects the control situation (siNT) where a drop in resistance (contraction) and recovery phase (to baseline levels) are observed; **green** reflects a decreased thrombin response (smaller AUC) as compared to control usually characterized by an attenuated drop (contraction) and/or improved recovery phase; **red** reflects an exaggerated thrombin response (bigger AUC) as compared to control usually associated with an increase in permeability. D—Quantification of area under the curve of the controls used in this analysis (NT and siABL2) (n = 18, in triplicate) ****P<0.0001 in Student’s T-test; E—Absolute endothelial resistance curves of HUVECs transfected with siABL2 and NT control siRNA upon thrombin stimulation (1U/ml) at 72h post-transfection for 3h (n = 18, in triplicate).
(EPS)

S2 Fig Raw endothelial resistance curves. A-O—Absolute endothelial resistance of HUVECs, transfected with the indicated siRNAs and NT control siRNA upon thrombin stimulation (1U/ml; arrow) at 72h post-transfection for 3h (n = 3).
(EPS)

S3 Fig Raw endothelial resistance curves. A-H—Absolute endothelial resistance of HUVECs, transfected with the indicated siRNAs and NT control siRNA upon thrombin stimulation (1U/ml; arrow) at 72h post-transfection for 3h (n = 3).
(EPS)

S1 Table Overview of the significant genes identified in the siRNA screen by comparing the drop in resistance induced by thrombin stimulation (1ml/U) and respective baseline values (n = 3). Hits are ranked by False Discovery Rate (FDR).
(DOCX)

S2 Table Overview of the significant genes identified in the siRNA screen by comparing the recovery level after stimulation by thrombin (1ml/U) and respective baseline values (n = 3). Hits are ranked by False Discovery Rate (FDR).
(DOCX)
