# Supplementary material for: A functional siRNA screen identifies RhoGTPase-associated genes involved in thrombin-induced endothelial permeability
Source: PLoS One. 2018 Jul 26;13(7):e0201231. doi: 10.1371/journal.pone.0201231 (PMC6062096; doi:10.1371/journal.pone.0201231)
Supplement: S1 Table — Hits are ranked by False Discovery Rate (FDR). (DOCX) [file pone.0201231.s004.docx]

**Supplemental Table 1** – Comparison between thrombin-induced drop in resistance versus baseline resistance values. Results ranked by FDR.

|  | **DROP VS BASELINE (1% HSA)** | | |
| --- | --- | --- | --- |
| **Gene ID** | **t-test** | **P value** | **FDR** |
| ARPC1B | -5,10181 | 0,0000175 | 0,004788 |
| RTKN | -4,61045 | 0,0000698 | 0,009566 |
| SRGAP2 | -4,02615 | 0,000355 | 0,026066 |
| FARP1 | 3,942613 | 0,000447 | 0,026066 |
| CDC42 | 3,919895 | 0,000476 | 0,026066 |
