# Supplementary material for: A functional siRNA screen identifies RhoGTPase-associated genes involved in thrombin-induced endothelial permeability
Source: PLoS One. 2018 Jul 26;13(7):e0201231. doi: 10.1371/journal.pone.0201231 (PMC6062096; doi:10.1371/journal.pone.0201231)
Supplement: S2 Table — Hits are ranked by False Discovery Rate (FDR). (DOCX) [file pone.0201231.s005.docx]

**Supplemental Table 2** – Comparison between resistance levels at 3h post-thrombin stimulation (recovery) versus baseline resistance values. Results ranked by FDR.

|  | **RECOVERY VS BASELINE (1% HSA)** | | |
| --- | --- | --- | --- |
| **Gene ID** | **t-test** | **P value** | **FDR** |
| ARPC1B | -7,07252 | 0,000000729 | 0,00002 |
| MLC1 | -6,15682 | 0,0000009 | 0,000123 |
| RTKN | -5,43656 | 0,00000679 | 0,00062 |
| RAPGEF2 | -5,05133 | 0,0000202 | 0,00138 |
| CNN1 | -4,87891 | 0,0000328 | 0,001666 |
| TRIO | -4,84116 | 0,0000365 | 0,001666 |
| MYO9B | -4,71122 | 0,0000526 | 0,002059 |
| Ppp1r12A | -4,64225 | 0,0000639 | 0,002187 |
| RACGAP1 | -4,55325 | 0,000082 | 0,002491 |
| RhoD | -4,51645 | 0,0000909 | 0,002491 |
| SEPT2 | -4,10701 | 0,000284 | 0,007082 |
| DNMBP | -3,99092 | 0,000391 | 0,008939 |
| TIAM2 | -3,91454 | 0,000483 | 0,010173 |
| VILL | -3,85929 | 0,000561 | 0,010985 |
| PAK7 | -3,65265 | 0,000982 | 0,017941 |
| STARD13 | -3,59159 | 0,001157 | 0,019812 |
| SYDE1 | -3,47207 | 0,00159 | 0,023673 |
| ARHGEF26 | -3,47729 | 0,001569 | 0,023673 |
| ARHGEF7 | -3,46009 | 0,001642 | 0,023673 |
| Cnksr1 | -3,33507 | 0,002281 | 0,028091 |
| CDC42EP2 | -3,32232 | 0,002358 | 0,028091 |
| SPATA13 | -3,3474 | 0,002208 | 0,028091 |
| RHOBTB2 | -3,36781 | 0,002093 | 0,028091 |
| PAK6 | -3,23628 | 0,002949 | 0,033668 |
| SOS1 | -3,22042 | 0,003072 | 0,033673 |
| ARHGAP45 | -3,20064 | 0,003233 | 0,034073 |
| ARHGEF5 | -3,14814 | 0,0037 | 0,037551 |
| CDC42EP5 | -3,11506 | 0,004027 | 0,038047 |
| CHN1 | -3,11995 | 0,003977 | 0,038047 |
| ARHGDIG | -3,09629 | 0,004224 | 0,038581 |
